# Endoplasmic reticulum stress and oncomir-associated chemotherapeutic drug resistance mechanisms in breast cancer tumors

**DOI:** 10.3906/biy-2010-62

**Published:** 2021-02-09

**Authors:** Leila MEHDIZADEHTAPEH, Pınar OBAKAN YERLİKAYA

**Affiliations:** 1 Department of Molecular Biology and Genetics, Faculty of Science and Letters, İstanbul Kültür University, İstanbul Turkey

**Keywords:** Endoplasmic reticulum stress, unfolded protein response, multidrug resistance, oncomir, breast cancer

## Abstract

Breast cancer, as a heterogenous malign disease among the top five leading causes of cancer death worldwide, is defined as by far the most common malignancy in women. It contributes to 25% of all cancer-associated deaths after menopause. Breast cancer is categorized based on the expression levels of cell surface and intracellular steroid receptors [estrogen, progesterone receptors, and human epidermal growth factor receptor (HER2)], and the treatment approaches frequently include antiestrogen, aromatase inhibitors, and Herceptin. However, the management and prevention strategies due to adverse side effects stress the patients. The unsuccessful treatments cause to raise the drug levels, leading to excessive toxic effects on healthy cells, and the development of multidrug-resistance (MDR) in the tumor cells against chemotherapeutic agents. MDR initially causes the tumor cells to gain a metastatic character, and subsequently, the patients do not respond adequately to treatment. Endoplasmic reticulum (ER) stress is one of the most important mechanisms supporting MDR development. ER stress-mediated chemotherapeutic resistance is very common in aggressive tumors. The in vitro and in vivo experiments on breast tumors indicate that ER stress-activated protein kinase R (PKR)-like endoplasmic reticulum kinase (PERK)- activating transcription factor (ATF4) signal axis plays an important role in the survival of tumors and metastasis. Besides, ER stress-associated oncogenic microRNAs (miRNAs) induce chemoresistance in breast tumors. We aimed to have a look at the development of resistance mechanisms due to ER stress as well as the involvement of ER stress-associated miRNA regulation following the chemotherapeutic regimen in the human breast tumors. We also aimed to draw attention to potential molecular markers and therapeutic targets.

## 1. Introduction

Amongst all the malignant diseases, breast cancer is one of the leading causes of cancer mortality in the women population worldwide, with 18.1 million new cases and 9.6 million cancer-related passing reported, according to the 2018 World Health Organization cancer case data. Breast cancer rate differs widely, ranging from 27/10^6^ (Africa and Central-East Asia) to 85–94/10^6^ (North America, Australia, and Western Europe) (Sancho-Garnier and Colonna, 2019). Most breast cancers occuring the breast tissue made up of hyperproliferation of the ducts that connect the lobules to the nipple. After being constantly stimulated by various carcinogenic risk factors, they are transforming into localized tumors or even metastatic carcinomas (Hosseini et al., 2019). Commonly therapeutic strategies for sophisticated breast cancer patients with faraway organ dissemination often are aforethought irremediable due to negative results. As a heterogeneous disease, according to molecular subtypes treatment approaches differ in breast cancer. Molecular attributes comprise activation of HER22 (HER2, encoded by *ERBB2*), activation of hormone receptors (estrogen receptor and progesterone receptor), and/or *BRCA* mutations. Treatment of breast cancer is multidisciplinary; it covers locoregional (surgery and irradiation) as well as systemic treat attempts. Systemic therapies contain anti-HER2 therapy for HER2-positive disease, poly (ADP-ribose) polymerase inhibitors for *BRCA* mutation carriers, the endocrinecure for hormone receptor positive patients, chemotherapy, bone stabilizing factors, and, nowadays immunotherapy (Harbeck et al., 2019).

Resistance to chemotherapy, which is considered the principal strategy in cancer treatment, constitutes serious obstacles in terms of the treatment effect and the survival process. It is thought that ability to adapt and rapidly become resistant to chemotherapy occurs due to changes in various biomarkers, with the inclusion of epigenetic alterations, gene transmutation and/or amplification, and miRNA expression. Taking into account tumor biology it is necessary to the enlightenment of complex changes, the interactions of intracellular checkpoints, and the underlying molecular mechanisms in a multidisciplinary setting.In this way, therapy concepts will be following a curative intent (Najjary et al., 2020).

Accumulating evidence indicates that one of theremarkable mechanisms that support MDR development is often mediated by the state of ER stress as wellas the actuationof the unfolded protein response pathways (UPR) within the intracellular endoplasmic reticulum whichknown as the quality control center of newly produced proteins. To maintain metabolic homeostasis, UPR regulates the capacity to handle stress and different pathways to block or encourage cell death. Recently, molecular mechanisms linking ER stress and durability to chemotherapy have been demonstrated in isolated tumors, and the “multistress resistance” phenotype hypothesis is advocated (Salaroglio et al., 2017).

Focusing on the mechanisms of resistance to chemotherapeutic agents due to ER stress and miRNA regulation in human breast tumors and alternative potential therapeutic targets to the responsible molecular markers has been amid in this review. 

### 1.1. Multidrug resistance 

Resistance to classical chemotherapeutics in cancer is defined as a phenomenon in whichtumor cells become tolerant of the pharmaceutical healer. As a form of intrinsic cellular insensitivity, acquired resistance to chemotherapeutic agents causes adverse impacts such asmetastasis, chemotherapy unsuccess, and eventually cancer-related death. Numerous mechanisms like epigenetic alterations and/or genetic aberration also various cellular and molecular mechanisms have been proposed to mediate intrinsically or acquired MDR in cancer cells (Wang et al., 2019; Mendes et al., 2020). Important clinical studies aimedat identifying potential MDR biomarkers, namely genetic variants, have been conducted to predict sensitivity levels in drug-selected model cancer cell lines (De Mattia et al., 2015; Zhang et al., 2019). It is estimated that drug impact is immensely mutable among patients and genetic variants comprise 20%–95% of the instability of drug-related response. The results of investigations may help guide future research and design individualized therapies in refractory cancer and tumor recurrence (Mukerjee et al., 2018).

According to Li et al. (2017), the MDR contraption is divided into seven sections. These are, respectively, (i) increasing drug efflux by membrane transporters as the primary transporters with ATP binding cassette transporters (ABC), (ii) decreased drug intake by solute carriers, (iii) blockage of apoptotic signaling through mutations in the p53 pathway oralteration in the expression level of B cell lymphoma (BCL) family proteins, (iv) **i**nduced drug metabolism, including subtraction bycytochrome P450 as well as glutathione S-transferase enzymes, (v) changing drug targets or hitting other targets and signaling pathways, (vi) miRNA andepigenetic regulation resulting in increased adaptation, and finally, (vii) alterations in the microenvironment due to hypoxia and cancer stem cell arrangement.

### 1.2. Innate and acquired drug resistance

#### 1.2.1. Innate resistance 

Natural resistance, which is generally described as innate resistance, occurs before the patient is exposed to medication and plays a vital role in drug efficiency. The medicinal action of the administered drug can be lessened by activation of the internal pathways that areused as a cellulardefense. As the most common causes of innate resistancein tumors; (i) preexisting genetic mutations that result in a reduced ability of cancer cells to respond to both chemo and target drugs, such as triple-negative breast cancer cells; (ii) presence of insensitive subpopulations, including cancer stem cells,developing adaptation with drug therapy; (iii) internal pathways preferred as a defense against ecological toxins (like anticancer remedy) (Huang et al., 2016).

#### 1.2.2. Acquired resistance 

Acquired drug resistance could be depicted by the fractional reduction of a drug’s anticancer activity after the chemotherapy treatment.Acquired resistance may develop due to (i) proto-oncogene activation; (ii) mutations or varying expression grade of drug targets; (iii) alterations in the tumor microenvironment (TME) after treatment. When acquired drug resistance makes progress in the tumor, to avoid a relapse in later stages of cure, the treatment plan should be redesigned accordingly (Wang et al., 2019).

### 1.3. Mechanisms of drug resistance 

**Figure 1:**
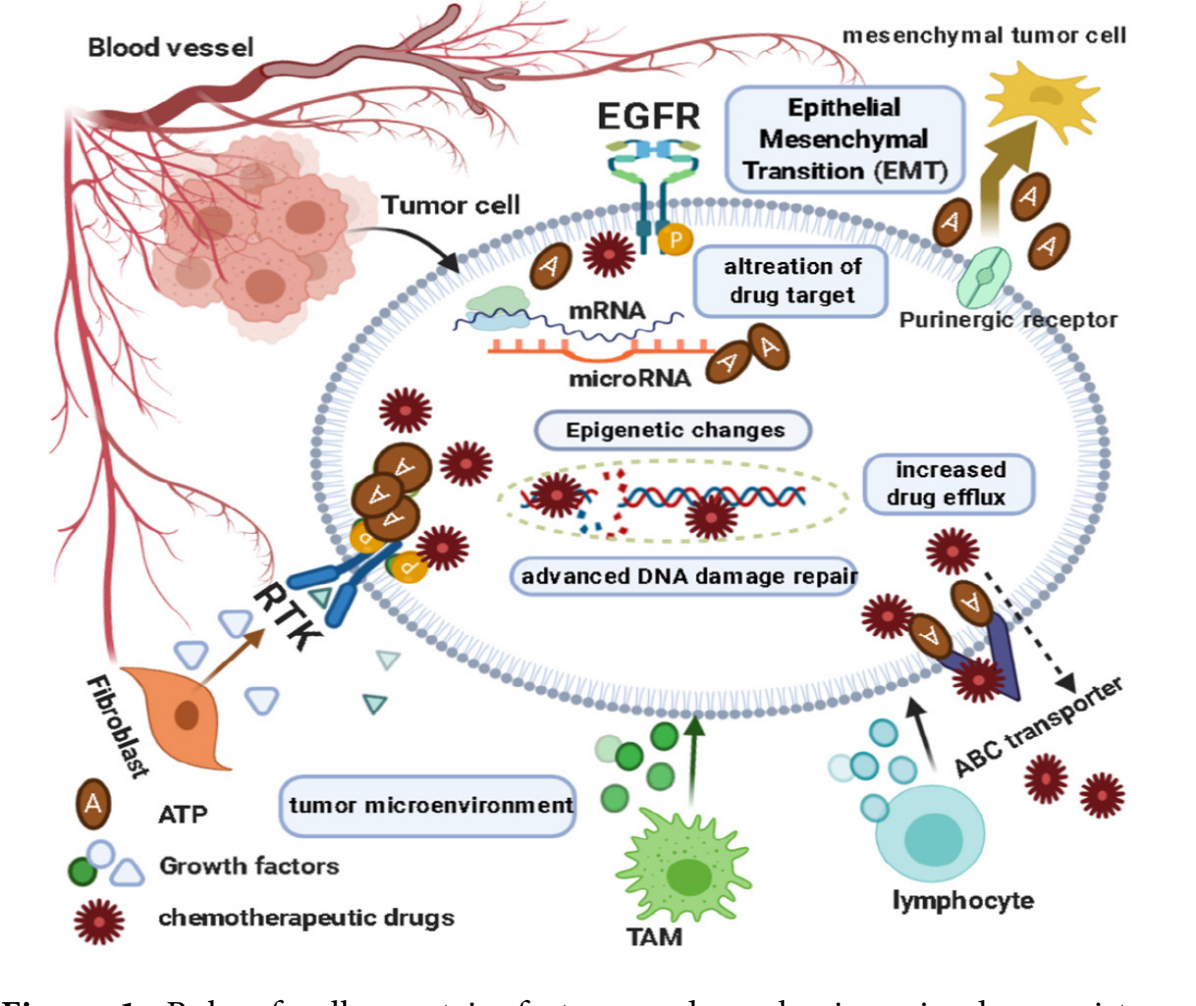
Role of cells, protein factors, and mechanisms in drug-resistance development in the tumor cell.

The importance of the molecular mechanisms of natural and acquired resistance categories, as shown in Figure 1, are examined is particularly important in terms of clinical results and the determination of therapy strategies. The clinical significance of the molecular contraptions of innate and acquired resistance categories which can be obtained over tumor progress and treatment is particularly crutial in determining clinical results and therapy strategies (Mendes et al., 2020). 

#### 1.3.1. Elevated efflux of drugs

Following drug intake into the cell, excretion of drugs through carrier proteins localized in the cell membrane has been accepted as the main cause of chemotherapy resistance.The ATP-binding cassette (ABC) transporter superfamily comprises transmembrane importers and exporter proteins. A variety of substrates, including lipids, sterols, metabolic products, and drugs, is translocating by these carriers. Overexpression of ABC transporters is often seen in tumors and multidrug-resistant cancer cells (Vasiliou et al., 2008; Wilkens, 2015). The roles of ABCG2, ABCB1, and ABCC1 proteins have been discussed in several reports in MDR acquiring patients. ABCG2, expressed on apical cell membranes, has a notable role in tissue protection against various xenobiotics and carries different endogenous and exogenous substrates, including chemotherapeutic agents such as mitoxantrone and various tyrosine kinase inhibitors, suggesting that ABCG2 is an important determinant of the pharmacokinetic properties of drugs (Toyoda et al., 2019). Balaji et al. (2016) have reported an increase in the mRNA levels of ABCC1 and ABCC3 by exposing breast cancer cells to doxorubicin (DOX), mitoxantrone (MXR), 5-flurouracil (5-FU). Besides, the increased drug sensivity have been exhibited in ABCC1 and ABCC3 knockdown model cell lines (Balaji et al., 2016). The receptor tyrosine kinase-like orphan receptor 1 (ROR1), which is largely expressed during embryogenesis but inhibited in normal adult tissue, is the upstream regulator of ABCB1. Recent studies have reported that ROR1 inhibition in breast tumors induces chemosensitivity by modulating MAPK/ERK and p53 signaling pathways (Fultang et al., 2020).

#### 1.3.2. Replacement of drug target

Conventional chemotherapy drugs slow down the proliferation of tumor cells and encourage cell death. Targeted therapeutic agents prevent cancer cell expansion by targeting the activity of particular proteins effective in tumor growth. At the same time, compared to traditional chemotherapythey minimize the cytotoxic effects in normal cells and therefore boost the success rate (Wang et al., 2019). The new therapeutic strategies are focused on multiple targets rather than a single one and inhibit the activation of target changes mechanisms. However, the crossinteractions of signaling pathways that affect the performance of administered drugs cannot be ignored (Cava et al., 2018). For example, in TNBC, AKT inhibitors are deactivated by the activation of receptor tyrosine kinases (RTKs) and therefore lose their effectiveness over time (Duncan et al., 2012). Human EGFR 2 (HER2) and epidermal growth factor receptor (EGFR) have a critical impact in the initiation and progression of BC. The inhibitors that target these receptor tyrosine kinases are considered to be among the most successful targeted drugs commonly used. The clinical and laboratory researches have highlighted the role of the crosstalk between EGFR, HER2 and other signaling pathways which is an important molecular mechanism underlying chemoresistance (Yamaguchi et al., 2014).

A recent computational in silico approach performed by Cava et al. (2018) identified the altered signaling pathways as prospective drug targets in BC subtypes. The results of this study can be summarized as follows: (i) liver X receptor-retinoid X receptor (LXR/RXR) activation signaling, as a noval probable drug target specific for luminal A; (ii) NAD biosynthesis II pathway, as main potential drug target is exclusive in HER2 positive BC; and finally (iii) aryl hydrocarbon receptor signaling pathway, specific to basal like subtype (Cava et al., 2018).

#### 1.3.3. Advanced DNA damage repair

The best part of chemotherapeutic agents such as cisplatin and 5-fluorouracil (5-FU) activates cell death signaling pathways in tumorsby provoking DNA damage. The DNA damage response (DDR) of cells touched by anticancer agents by reducing the DNA repair mechanisms and effectiveness of drugs, may lead to the progress of MDR (Wang et al., 2019). Targeting DNA repair mechanisms is considered as an important therapeutic approach in breast tumors especially for those characterized by a “BRCAness” phenotype including the “triple-negative” subtype. BRCA1/2 has a significant effect on homologous recombination (HR), which is the fundamental mechanism in the repair of double-stranded DNA breaks (DSBs). BRCA mutated breast cancer patients exhibit different clinicandpathological characteristics related to indigenous deficiency in HR DNA repair (Paluch-Shimon and Evron, 2019). The cisplatin-based neo-adjuvant clinical trials in TNBC showed that homologous recombination deficiency (HRD) is indispensable for cisplatin sensitivity. The frequency of HRD is low in response to endocrine therapy in HER2-overexpressing (HER2+) cell line (Manié et al., 2015).

#### 1.3.4. Senescence break

Senescence is a cellular process defined by the cessation of cell division by age. It can be induced as a response to stress conditions such as oncogenic activation, radiation, genotoxic drugs, metabolic disruptions, DNA damage, and encourage the prevention of irreversible malignant cell growth. The lysine 9 trimethylation of p16INK4a, p21CIP1, p53, and histone H3 (H3K9me3), which are known as critical regulators of stem cell functions, have tremendous effects on tumor aggressiveness and clinical treatment success representing the basic signaling components of the aging mechanism simultaneously (Milanovic et al., 2017). Chemotherapeutic drugs are highlighted to affect many cellular responses including activation of age-promoting signals in the cancer cell and tumor microenvironment. Therapy-related aging is a condition that can lead to the disappearance of tumor cells by stimulating immune surveillance and can be a source of chronic inflammation and contribute to MDR development (Demaria et al., 2016).

Experimental studies are showing that the treatment of breast cancer patients with anthracycline and alkylating agents causes p16INK4a dependent, telomere-independent irreversible cellular aging, and the senescence-associated secretory phenotype (Sanoff et al., 2014). The feature of aging mechanisms thrivesamong different tumor cells. This may partially elucidate the variability in chemoresistance and treatment failure rates observed in clinical applications (Demaria et al., 2016).

#### 1.3.5. Epigenetic changes

Increasing evidence shows that epigenetic modifications also participate in MDR mechanisms. Epigenetic alterations are defined within the scope of DNA methylation, chromatin remodeling, histone modification, and noncoding RNA-related mutations, which do not involve sequence alterations in the DNA structurebut can be transferred from cell to cell. Following chemotherapy, tumor cells show a notable increase or decrease in the expression levels of drug carriers (ABCB1, MDR), DNA repair proteins, histone modifiers, or proapoptotic genes, as a result of epigenetic changes (Assaraf et al., 2019). Recent studiessuggest that chemoresistance is gradual, multifactorial, and includes genetic and epigenetic alterations. The mutations of epigeneticsmechanism regulatory genes, including the ten eleven translocation enzymes (TET), DNA (cytosine-5)-methyltransferase 3A (DNMT3A) and polycomb repressive complex 2 (PRC2) cause the disorganization of CSC pathways and managing cellular reprogramming and finally can induce the acquired chemo-resistance (Ponnusamy et al.,2019). In basal-like breast cancer, a significant increase in bromodomain-containing protein 4 (BRD4), lysine-specific demethylase 5B (KDM5B), and enhancer of zeste homolog 2 (EZH2) activities was detected in tumor cell populations after treatment with MEK and PI3K/mTOR inhibitors. Moreover, it has been observed that the genes involved in the SWI/SNF chromatin remodeling such as AT-rich interactive domain-containing protein 1A ARID1A, ARID1B and ARID2 are mutated in chemo-resistant patients (Risom et al., 2018). According to preclinical findings poor prognosis results with overexpression of lysine demethylase 6B (KDM6B) and insulin-like growth factor-binding protein 5 (IGFBP5) especially in luminal subtypes is related to an epigenetic switch from H3K27me3 to H3K27Ac at the promoter region of GFBP5 gene (Wang et al., 2018).

#### 1.3.6. Tumor heterogeneity

Tumor heterogeneity is under review in four main categories as genetic heterogeneity, cell type heterogeneity (cancer cells, stromal cells, immune cells, etc.), metabolic heterogeneity, and transient heterogeneity in dynamic tumor progression. Uncontrolled changes in genetic, epigenetic, and phenotypic modifications, including microenvironment and metabolic imbalance, lead to breast tumor formation. Breast tumors are characterized by cell-to-cell, spatial and provisional heterogeneity features. Sequencing analyzes prove that serious changes can occur after neoadjuvant therapy and genomic modification. However, it is a complicated situation, whether the observed tumor changes externalize treatment-related clonal evolution rather than preexisting intratumor heterogeneity (ITH) (Baliu-Piqué et al., 2020). Whole exome sequencing of primary breast tumors achieved by Caswell-Jin et al. (2019) exhibited significant heterogeneity and large clonal replacement and polyclonal resistance occurring in a small tumor fraction. Finally, high resistance aberration rates in these tumors were noted throughout treatment with chemotherapy and HER2-targeted therapy (Caswell-Jin et al., 2019).

#### 1.3.7. Tumor microenvironment

Tumors cannot be defined as homogeneous groups of cancer cells. It encompasses a variety of cell types and extracellular matrix (ECM) that cooperate with all aspects of the cancer hallmarks.Immune cells, transformed ECM, reprogrammed fibroblasts, soluble factors, signaling molecules, immune suppressive cells and blood vessels play substantial roles in the growth, inhibition of apoptosis, angiogenesis, and drug resistance of solid tumors in a coordinated manner within the tumor microenvironment (TME). Exosomes as nanovesicles are promising in novel treatment strategies by establishing the communication scheme between TME and surrounding cells. The changes caused by chemotherapy in TME have the potential to induce the development of resistance and decrease in drug efficacy by contributing to the adaptation of cancer cells (Deepak et al., 2020). Upon the negative changes in the microenvironment, tumor cells initiate the adaptation process and activate various signaling cascades like mTOR, NF-KB, AKT (Qu et al., 2019) .

Although tamoxifen is the most efficient in current approaches for estrogen receptor α positive (ERα+) BC, which make up the maximal proportion of BC patients, development of resistance to tamoxifen in connection with metastatic recurrence occurs in most patients during the treatment. Gao et al. (2020) described the molecular mechanisms underlying the contribution of TME in MDR progress by designing a single-cell RNA sequencing in Luminal A BC cells. Their finding has revealed a novel subset of tumor-related fibroblast (CD63^+^CAF) in theTME, which downregulates ER*α* and PTEN expression in model cell lines by means of exosomal miR-22, accordingly provoke tamoxifen resistance (Gao et al., 2020).

#### 1.3.8. Epithelial-mesenchymal transition

Mammary gland development, glandular regeneration, normal morphogenesis and organogenesis have been attributed to epithelial-mesenchymal transition (EMT) programs. The activation of EMT in transformed breast epithelial cells increases their ability to achieve invasive and metastatic behavior, as well as the emergence of chemotherapy-resistant breast cancer stem cells (BCSCs) simultaneously (Gooding and Schiemann, 2020). Tamoxifen-resistant cells acquire mesenchymal-like phenotypes mediated by upregulated expression of mesenchymal markers (TWIST1, SNAIL, Vimentin, N-cadherin, fibronectin, ZEB2), downregulated expression of epithelial markers (E-cadherin and miR-27b). Besides, the increased activity of the glycogen synthase kinase (GSK)-3beta and nuclear factor (NF)-kappaB signaling in the regulation of Snail-mediated E-cadherin also notable. The levels of methylation in the miR-27b promoter region also takes place during EMT (Kim et al., 2009; Li et al., 2016). Invasive breast cancer subtypes acquire EMT phenotype after over activation of the TWIST1/AKT signaling axis, leading to increased migration, invasion, and resistance to paclitaxel (Cheng et al., 2007). According to Gupta et al. (2009), EMT was induced by silencing transformed telomerase activity and shRNA-mediated E-cadherin expression in Ras mutant HMLER breast cancer cells. As a result, they reported that the population of CD44^+^/CD24^-^ cell numbers increased and these cells displayed mesenchymal cell formation, tumor formation, and metastasis compared to epithelial phenotypic cells, and most importantly, EMT supported drug resistance mediated by CSC-like markers (Kong et al., 2011). A recent study with the exposure to sublethal doses of chemotherapy showed that the migration capacity of BCSCs is uninfluenced while the sphere formation potential of cells are increased and EMT-associated gene expression at the mRNA level are altered significantly (Li et al., 2020).

### 1.4. Signaling pathways associated with improvement of multidrug resistance in breast cancer

Triple-negative breast cancer (TNBC) is the virulent subtype of breast cancer due to its offensive nature and deficiency of therapy options. Though optimal protocols for TNBC therapy have not been established, chemotherapy remains the onlysystemic treatment alternative. However, it is observed that patients develop resistance to frequently administered drugs. While anthracycline and taxane-based regimes are the basis for TNBC heal, platinum-based chemotherapies have exhibited controversial results in the metastasis process. Consequently, immense attempts have been done into clarifying the contraption of TNBC chemoresistance, aimed to recognize novel molecularobjects. Based on circumstantial interaction of the TME, CSCs, drug efflux, and mass tumors the progress of TNBC chemoresistance is sophisticated (Yuan et al., 2016).

The stromal microenvironment of tumors includes cancer stem cells (CSCs) and their heterogeneous progeny. Transforming growth factor-beta (TGF-β) signaling as an immunomodulator, without doubt, assists proliferation, angiogenesis, EMT, metastatic distribution, and chemoresistance. Epithelial and carcinoma cells undergo a partial or complete epithelial-mesenchymal transition (EMT) in response to transforming growth factor- β (TGF-β). Katsuno et al. (2019) have been selected HMLE, NMuMG and H-Ras-transformed carcinoma cell populations to examine the molecular process of stem cells, EMT phenotype, and drug resistance development, related to time-dependent TGF- β exposure. Their results have shown that short term (9–12 days) also long term (24 days) exposure induces reversible EMT and stable EMT, respectively. While reversible EMT strongly increases the emission and metastasis, the stabilized EMT phenotype cause to steady enhanced stem cell production which, is accompanied by (mTORC1/mTORC2) signaling pathways, resulting in anticancer drug resistance (Katsuno et al., 2019).

TRIM32 with an E3 ubiquitin ligase activity, known as the regulator of NF-κB signaling by ubiquitination of protein inhibitor of activated STAT Y (Piasy). TRIM32 dysregulation has been implicated in various human cancers. To examine the effect of TRIM32 on chemo-resistance development of breast epithelialcells (MCF-10A), and other ductal carcinoma, and invasive ductal carcinoma cell lines, CCK8 assay and Western blot analysis were employed. Their findings demonstrated the decreasing TRIM32 expression in MCF-10A normal breast epithelial cells. The overexpression of TRIM32, upregulation ofIAPanti apoptotic protein, upregulation of p-p65, p-IκB levels as NF-κB signaling activators and downregulation of cell cycle inhibitors (p21 and p27) have been resulted in cell viability and chemo-resistance in MCF-7cells. Conversely, in T47D cells downregulation of TRIM32, diminished cisplatin resistance and cell viability. These data suggest that NFκB might be a potential target for breast cancer initiation progress and chemoresistance (Zhao et al., 2018). Abnormal JAK/STAT signaling governs a series of processes such as survival, proliferation, tumorigenesis, spreading, angiogenesis, immune suppression as well as apoptosis. While IL6 and IL8, which are among the genes identified to support TNBC cell division, have a minor effect on estrogen receptor positive tumor cells showed by in vitro and in vivo studies. Conversely, simultaneous inhibition of IL6 and IL8 has been reported to reduce proliferation, promote apoptosis, and increase paclitaxel sensitivity (Hartman et al., 2013).

Dipeptidyl peptidase-4 (DPP-4) inhibitors are defined as an oral hypoglycemic class that prevents inactivation of glucagon-like peptide 1 (GLP-1) by blocking the dipeptidyl peptidase-4 enzyme. In vivo and in vitro studies showed the association between DPP-4 inhibition and the increase of ABC transporters. Li et al. (2020) investigated the effects of DPP-4 suppression on chemoresistance in mouse and human breast cancer cell lines using specific shRNA and DPP-4 inhibitor KR62436 in 4T1 cells. Their findings suggest that there was no significant change in the expression level of P-gp and ABCG2 in 4T1 cells treated with the inhibitor. Whereas the levels of P-gp and ABCG2 increased significantly in 4T1 cells treated with doxorubicin (DOX) or the inhibitor and DOX combined therapy. Finally, increased expression of ABC transporters have been reported as a result of DPP-4 deficiency, and that 4T1 cells acquired susceptibility to chemotherapy-induced apoptosis, mediated by the CXCR4/mTOR axis or the TGF-β signaling pathway (Li et al., 2020).

Exosomes, subderivatives of extracellular vesicles surrounded by a lipid bilayer membrane approximately 100 nm in diameter, are considered to support the development of tumor drug resistance. Lv et al. (2014) used the ultracentrifugation method to form MCF-7/DOX (doxorubicin-resistant) cells from drug-sensitive wild-type MCF-7 cells. By removing the exosomes from the cell supernatant they have been accessed the P-gp expression levels with flow cytometry. This study emphasizes that P-gp transmission from exosome-mediated resistant cells to parental drug-sensitive cells may be a significant mechanism of drug resistance transfer. Growing evidence connect the vital role of exosomes with angiogenesis, migration, avoidance of immune cell attacks, metastatic niche formation, MDR, and cancer progression. There is an increasing interest in validating and incorporating them into clinical applications in recent years (Osaki and Okada, 2019).

In response to ATP treatment in breast cells, sex determining region Y-box 9 (SOX9) is known as a chief regulator of extracellular ATP signaling. In vitro invasion and migration assays in a recent study demonstrated that after ATP treatment in MDA-MB-231, BT-549, T47D, and MCF10A breast cell lines, ATP-IL-6-SOX9 signaling stimulate breast samples aggression and chemoresistance.CEACAM5/6 defined as potential target genes of SOX9, mediate ATP-induced invasion, and also ABCB1 and ABCG2 to mediate ATP-driven chemoresistance. According to the obtained results SOX9 could not be ignored in novel treatment strategy approaches (Yang et al., 2020).

The PI3K/Akt/mTOR pathway is highlighted as one of the main causes of cancer cell resistance to antitumor therapies. Dysregulation of this signaling pathway is involved in a wide variety of cancer hallmarks enhancement like proliferation, metabolic reprogramming, genomic instability, immune response regulation, survival, and motility. Despite the detailed clinical studies and the presence of effective PI3K/Akt/mTOR inhibitors, the underlying cellular events are not yet known, unfortunately (Ortega et al., 2020). 

Identification of MDR-related genes and pathways in hormone receptor positive breast cancer cells performed by KEGG analysis showed that target genes were supported in several pathways, with the inclusion of tight junction, calcium, CAM, or Wnt signaling pathways. Besides to deterioration of homeostasis, interleukin-6, estrogen, and nomegestrol acetate affect chemoresistance in breast tumors (Yang et al., 2018).

With the three FDA approved selective CDK4/6 inhibitors: abemaciclib, palbociclib, and ribociclib, limited success has been achieved as a result of intrinsic or acquired resistance. One of the main factors of uncontrolled cell proliferation and CDK4/6 inhibitor resistance frequently seen in breast tumors is the deregulation of the cyclin D-CDK4/6-INK4-RB axis, upstream response, and downstream bypass mechanisms (Li et al., 2020).

Numerous evidence has shown Exosomes’ main role in BC advancement as intercellular communicative vectors. Regulation of drug efflux and metabolism, activation of bypass signaling or prosurvival pathways, epithelial-mesenchymal transition, promotion of DNA damage repair, stem-like characteristic, remodeling of the microenvironment, and drug resistance (Dong et al., 2020).

Recent work with O’Brien et al. (2020) in PIK3CA mutant and wild-type ER^+^/HER2^-^ cell lines revealed that either a direct loss of Rb or loss of dependence on Rb signaling confers cross-resistance to inhibitors of CDK4/6, while PI3K/mTOR signaling remains activated. in the absence of continued CDK4/6 inhibitor, Treatment with the p110α-selective PI3K inhibitor, alpelisib (BYL719), entirely blocked the progression of acquired CDK4/6 inhibitor-resistant xenografts (O’Brien et al., 2020).

### 1.5. Molecular mechanisms of the unfolded protein response

**Figure 2:**
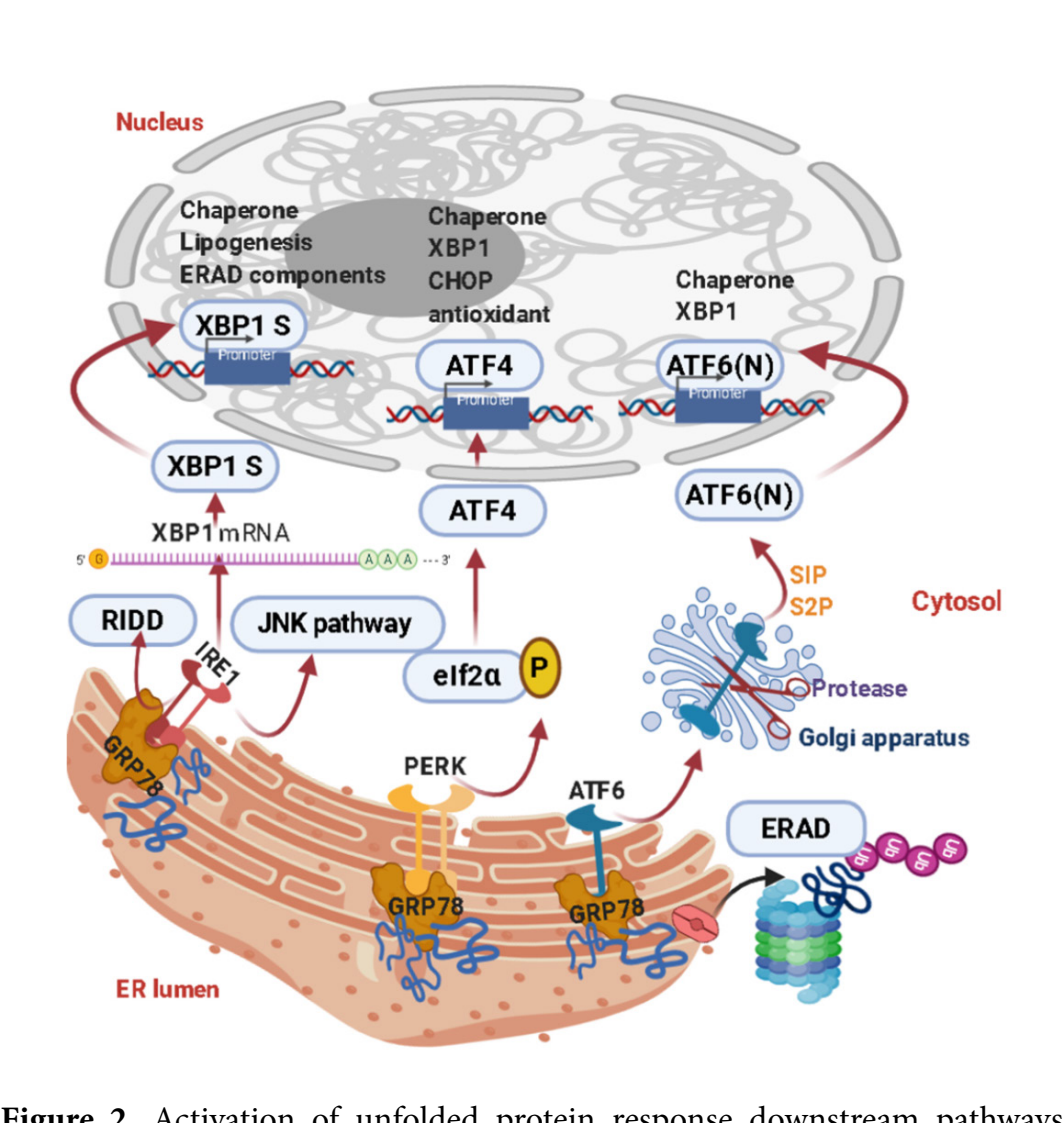
Activation of unfolded protein response downstream pathways under ER stress condition.

ER is defined as the site where newly produced proteins are folded and subjected to posttranslational alterations before being sent to the Golgi apparatus for their final integrity. All proteins associated with the plasma membrane, including ABC transporters, follow this process. Each stage of protein modification agreeably folded proteins, and unfolded/misfolded polypeptides, are continuously inspected by the ER quality control system (ERAD/ERQC) in the ER lumen (Jiang et al., 2020). UPR is crucial to tailoring the ER folding capacity of cells under undesirable conditions such as nutrient and oxygen deprivation. PERK, ATF6, andinositol-requiring enzyme 1 (IRE1) are three main sensors, first identified in mammals in the late 1990s, to control the UPR process localized to the ER membrane. Under normal conditions, these stress sensors are kept ineffective owing to the ER chaperon, GRP78/BiP. However, separating from GRP78/BiP transmembrane proteins due to the developing stress, allowing them to be activated (Figure 2). At this point, the aim is to induce the expression of chaperones localized in the lumen and to temporarily reduce protein synthesis. A triggered UPR response offers the opportunity to restore ER capacity by rebalancing the protein load and folding (Hotamisligil and Davis, 2016).

Under normal conditions, the initial UPR signal begins with the activationand homodimerization of IRE1, PERK, and ATF6. IRE1α is the protein responsible for the initiation of the UPR response by dissociating from BiP in the ER lumen, is autophosphorylated, and induces the cut of the 6-basepair part of the XBP1 mRNA. Thus, the active XBP1 protein, a transcription factor, initiates the transcription of ER lumen chaperones and inflammatory genes. Autophosphorylation of IRE1α also provides activation of jun N-terminal kinase (JNK), which is involved in stress response. JNK is involved in the induction of caspase 12, which is considered a biomarker in ER stress. PERK, the second transmembrane protein involved in ER stress, becomes autophosphorylated after BiP separation and ensures that its subtarget, eukaryotic translation initiating factor 2 α (eIF2α), is phosphorylated. With this inhibitory phosphorylation of eIF2α, translation is suppressed and the cell cycle stops. PERK also takes part in the activating phosphorylation of ATF4 protein, which is a transcription factor of CCAAT-enhancer-binding protein homologous protein (CHOP). Finally, the activation of the ATF6 pathway is achieved by its translocation to the Golgi cisternae and then to the nucleus. ATF6 is a transcription factor that initiates the expression of genes encoding enzymes disposed of for protein folding and ER-resident chaperones (Hotamisligil and Davis, 2016; Hillary and Fitz Gerald, 2018).

#### 1.5.1. Multidrug resistance-associated with endoplasmic reticulum stress in breast cancer

**Figure 3:**
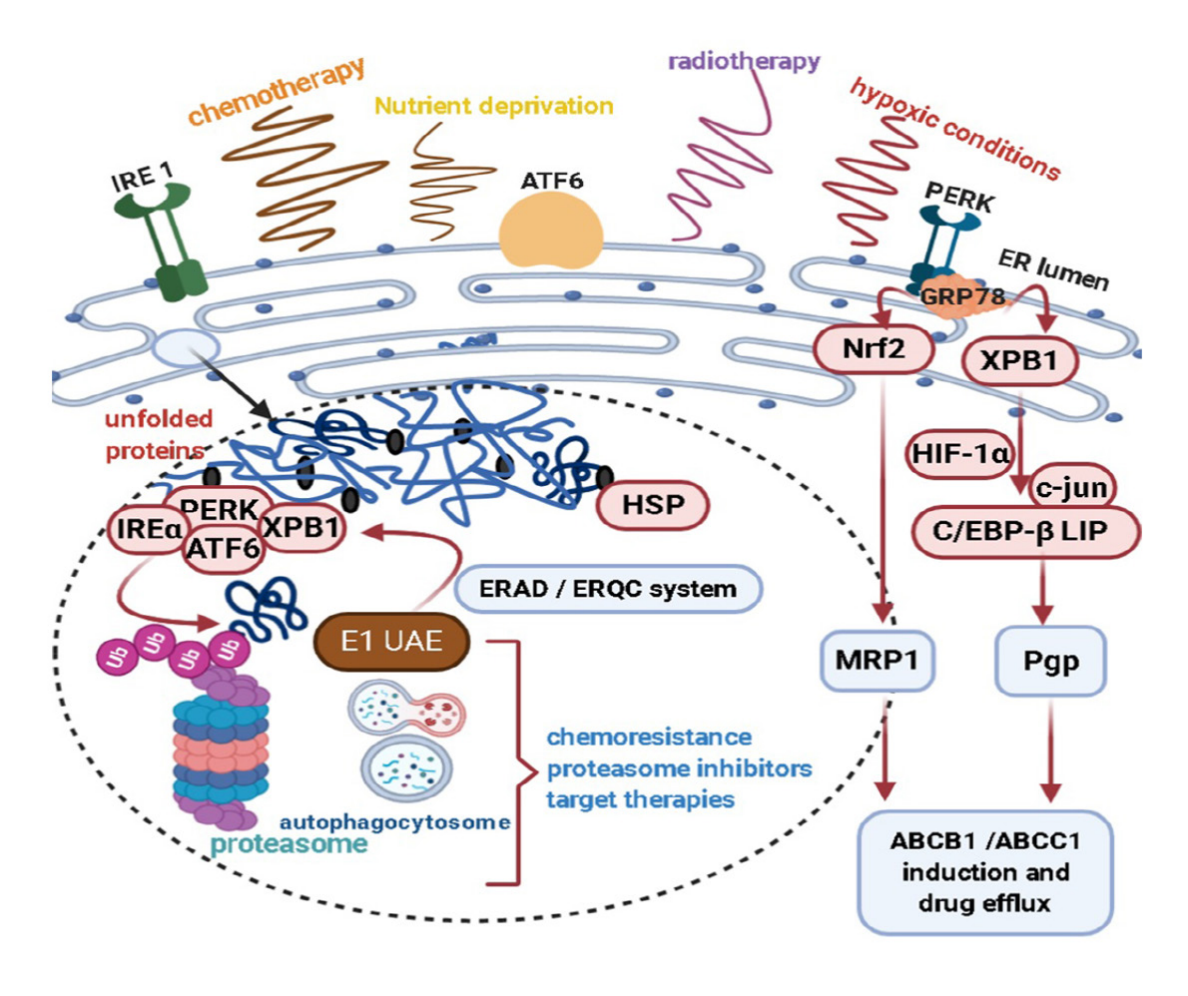
Altered endoplasmic reticulum function can lead to multidrug resistance.

As shown in Figure 3, inconvenient peripheral circumstances like hypoxic conditions, nutrient deprivation, irradiation, and chemotherapy cause a series of unfolded/misfolded proteins to collect in the ER lumen. The elimination of unfolded polypeptides is driven by ubiquitination, proteasomal/lysosomal degradation, or autophagy systems (Li et al, 2020).

Along with the initiation, migration, differentiation, metabolic changes, and adaptation processes to environmental stresses in tumor cells a remarkable amount of proteins are not properly folded. In these challenging conditions, the adaptive arm of unfolded protein response (UPR) capability and ER stress response (ERSR) regulation is critical for cancer cell survival, depletion of tumor suppressors, or activation of oncogenes and chemo-resistance. (Avril et al., 2017). Recent studies emphasize the critical role of UPR activation and chemotherapy resistance in many subtypes of breast cancer (Table). 

**Table:** Table. Cellular models highlighting the importance of chemoresistance development in BC associated with ER stress and UPR sensors.

Breast cancer subtype	Cell line	Therapeutic agent	Comments	Ref.
TNBC/ (mesenchymal stem like, MSL) TNBC/ (mesenchymal, M)Normal epithelial	MDA-MB-231BT-549HBL-100	Betulinic acid (BA) treatment	c-Myc-mediated glycolysis, apoptosis and drug resistance occurrence as a result of GRP78 Overexpression and PERK activation	Zheng et al., 2019
Normal epithelial luminal AHER2 positivehuman and murine TNBC cancer cells	MCF10A MCF7SKBR3 T47DMDA-MB-231 murine mammary cancer JC cells	Doxorubicin treatment and lysosome/ proteasome inhibitors	Suppression of C/EBP-β LIP degradation arises ER-dependent apoptosis, Pgp downregulation, DC/CD8+T-lymphocytes response reactivation and overcome resistance to drug.	Salaroglio et al., 2018
Basal like TNBC	SUM-159PTMDA-MB-436 MDA-MB-468	Docetaxel /NOS-inhibitor (L-NMMA) cotreatment	ATF4, CHOP pASK1 / JNK proapoptotic-mediated ER stress response, caspases 3 / 9 cleavage promotion and withdrawal of resistance.	Dávila-González et al., 2018
Luminal ABasal like TNBC	MCF-7MDA-MB-231	Taxol/Betulinic acid cotreatment	Enhancing chemo-sensitizing effects of taxol via ER stress-mediated cell cycle arrest at G2/M checkpoint induced by BA.	Cai et al., 2018
Luminal AHER2 positiveNormal epithelialTNBC	MCF7 T47D SKBR3MDA-MB-231 MDA-MB-468 MCF10A	Tunicamycin (Tm), MKC8866: selective IRE1 RNase inhibitor, Paclitaxel	IL-6, IL-8, CXCL1, GM-CSF, TGFβ2 protumorigenic factors production through IRE1 RNase activity. Increase efficacy of chemotherapeutics with the support of the IRE1 RNase suppression approach.	Logue et al., 2018
HER2 positive (ER+)	Rat DMBA-induced mammary tumors	Isoflavone genistein (GEN) during tamoxifen (TAM) therapy	Lifetime GEN intake reduces de novo resistance to TAM, compared with postdiagnosis GEN groups. Either lifetime or adult GEN intake inhibits TAM resistance and reduces local mammary cancer recurrence.	Zhang et al., 2017
Luminal A (sensitive)Luminal A (resistant)	MCF-7MCF-7/Dox	Nelfinavir (NFV), doxorubicin (DOX) cotreatment	Increased UPR-transducers (Grp78, p-PERK, p-eIF2α, and ATF4); and death sensors (CHOP & TRIB-3). Increased AKT level is linked to therapeutic resistance.	Chakravarty et al., 2016
Luminal A	T47D	Estrogen	Overexpression of ER chaperones such as BiP (GRP78 / HSAP5), p58IPK, calreticulin, leads to survival, angiogenesis, and resistanceto chemotherapy.	Andruska et al., 2015
Luminal A (resistant)	MCF7-TAMR T47D-TAMR	STF-083010 /TAM cotreatment	Reduced sensitivity to TAM via XBP1s / p-IRE1overexpression, STF-083010 Leading to IRE1-XBP1regulation and restored tumor sensitivity.	Ming et al., 2015
Luminal A (sensitive)Luminal A (resistant)	MCF7/LCC1 (antiestrogen-sensitive LCC1) MCF7/LCC9 (antiestrogen-resistant LCC9)	Selective ER downregulator; Fulvestrant (Faslodex; ICI 182780; ICI)	Inhibiting of antiestrogen-mediated UPR activation and autophagy, as critical resistance inducers. Overcoming chemoresistance by means of ER-α knockdown.	Cook et al., 2014

When it comes to chemoresistance, the role of UPR in counteracting MDR is controversial. In different breast tumor subtypes, UPR has been reported to support the development of resistance to radiation therapy, paclitaxel, tamoxifen, cisplatin, vinca alkaloids, doxorubicin, microtubule-interfering therapeutic agents and histone deacetylase (HDAC) inhibitors. On the other hand, UPR has been observed to induce cell death in other treatment approaches such as bortezomib, pan-peptidylarginine deiminase lapatinib/obatoclax combination (McGrath et al., 2018). In vitro and in vivo studies in this section highlight the UPR role in cell fate via both promoting and/or suppressing susceptibility to drug therapy and cell death. Studies show that TM9SF4 knockdown in adriamycin-resistant MCF-7 cells, which displays increased expression in cancer patients with chemoresistant tumors, triggers and increases ER stress level via upregulating ER stress markers and initiating the subsequent apoptosis cascade (Zhu et al., 2019). Vielanin K(VK) and doxorubicin (DOX) cotreatment in MCF-7/WT, MCF-7/MDR cells promotes JNK protein phosphorylation and activates IRE1α-TRAF2-JNK pathway, which in turn lead to mitochondrial apoptosis and increased sensitivity to chemotherapeutic drugs. Besides, IRE1α and JNK siRNA transfections weakened combination treatment-induced apoptosis. (Zhang et al., 2020). The effect of GRP78 expression as the direct interacting target on drug resistance substantially includes the reduced impact of drug-induced apoptosis. In this respect Xie et al. (2016) showed that the ectopic expression of GRP78 in MDA-MB-231 and MCF-7 wild-type and gemcitabine-resistant MDA-MB-231 and MCF-7 tumors altered drug sensitivity. Recently, molecular mechanisms linking ER stress and chemoresistance have been elucidated in different tumors. Emerging evidence suggests that cells that adapt to survive under severe ER stress conditions simultaneously acquire resistance to ER stress and chemotherapy. Gradual applications were applied to tumor cells with dissimilar ER stress inducers such as brefeldin A, tunicamycin, and thapsigargin. Cells adapted to ER stress gain the MDR phenotype, boost PERK expression, and PERK-linked Nrf2/MRP1 signal axis (Wu et al., 2018). Drugs that provide acquired resistance to ER stress can activate UPR-related genes such as P-gp, MRP1, MRP2, MRP3, MRP5 (multidrug resistance-associated proteins), and BCRP. Therefore, it is worth noting that ER stress sensors can be MDR inducers (Salaroglio et al., 2017). For instance, functional studies on TM9SF4 belonging to the transmembrane 9 protein (TM9SF) family describe that TM9SF4 is effective in cell adhesion, phagocytosis, and autophagy. Findings demonstrated that shRNA-mediated TM9SF4 silencing increases ER stress by causing misfolded protein accumulation in adriamycin-resistant MCF-7 and gemcitabine-resistant MDA-MB-231 breast tumorscompared to wild-type cells and may result in apoptotic and necrotic cell death activation.Based on the findings, it emphasizes that TM9SF4 may be a possible target therapy strategy to overcome the chemoresistance barrier in breast cancer treatment (Zhu et al., 2019).

### 1.6. Oncomirs and multidrug resistance

MicroRNAs are transcribed from the intron or exon regions encoding proteins on the genome and from DNA sequences in regions that do not encode a protein. Indeed, they are omnibus RNA molecules that do not translate protein, only play a major role in normal biological processes such as cell proliferation, differentiation, and death (Shenouda and Alahari, 2009). Due to their presence in blood circulation, their roles in many diseases, especially cancer, have been investigated. Considering the expression changes of miRNAs in tumor tissues and the mRNAs they target, emerging evidence suggests that these nonencoding proteins and small RNAs will provide a significant opportunity in the early diagnosis of cancer and the development of therapeutic agents. miRNAs, accepted as oncomirs when altered in tumor tissue, represent different levels of expression in cancer cells.Accumulating evidence support that various oncomirs play a vital role in the immunopathogenesis of breast cancer as well as in the development of chemoresistance, so they are considered promising as valuable prognostic biomarkers and therapeutical targets in distinct types of cancer (Hemmatzadeh et al., 2019). Various miRNAs, especially miR-122, miR-34c, let-7, miR-183, miR-200c, miR-203, and miR-16 affect cancer pathobiology by focusing on cellular and molecular targets important in breast tumor development. It is known that they can induce genomic instability by triggering DNA damage response. Besides, as compared to normal stem cells,miRNAs have diverse expression profiles in breast cancer stem cells, especially miR-200b-200a, miR-200c-141 also miR-183-96-182 have significantly lessened expression levels (Jafari et al., 2018).

#### 1.6.1. Biogenesis and function of microRNAs

**Figure 4:**
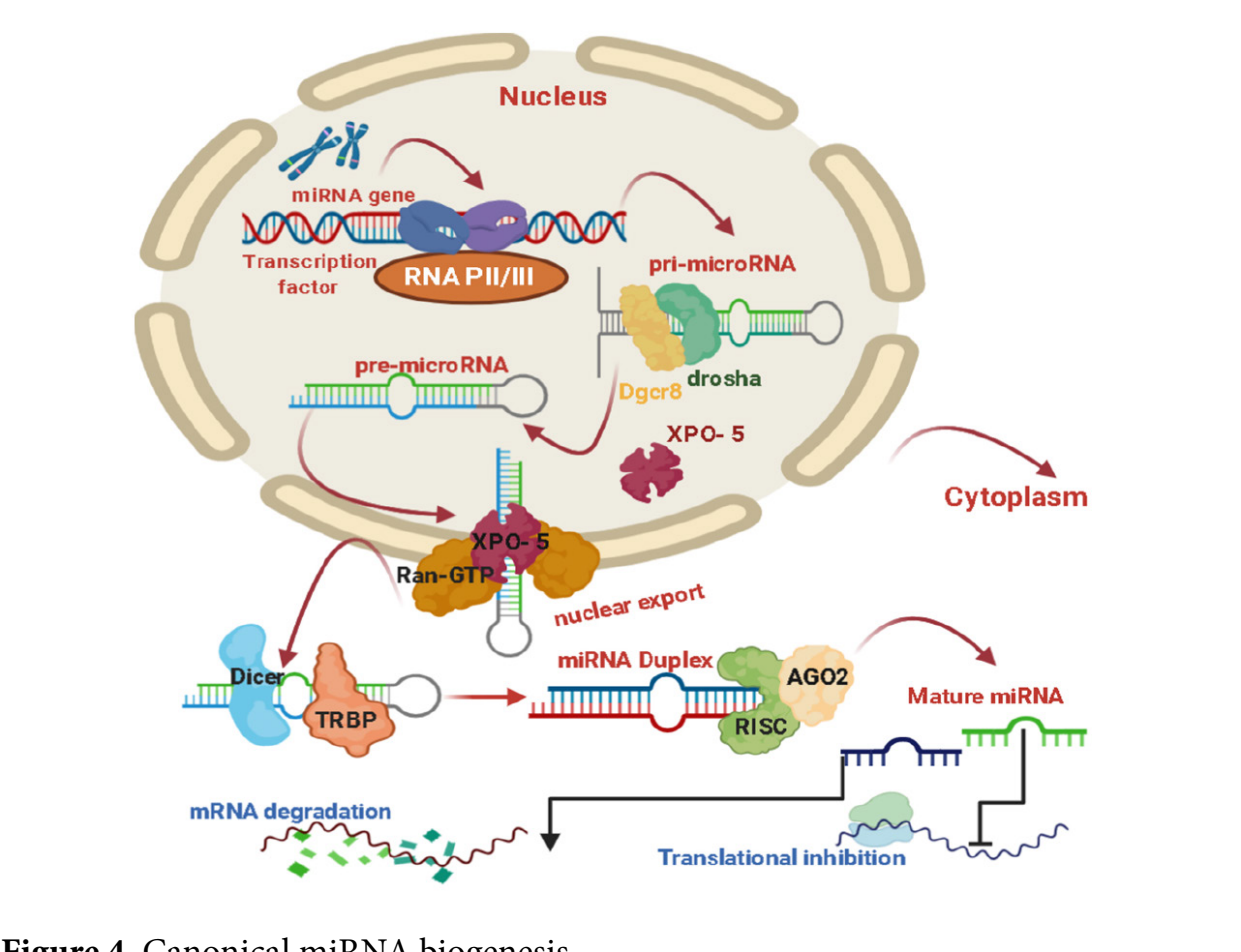
Canonical miRNA biogenesis.

MicroRNAs (miRNAs), with approximately 22 nucleotides in length, is a small set of noncoding RNAs that have a prominent role in regulating gene expression.When they bind to their complementary mRNAs, they induce translational silencing or disruption of the target. miRNAs are encoded by intergenic, intronic, or exonic regions of other genes, as in Figure 4, and transcribed as a long primary miRNA (pri-miRNA). It is processed into the precursor miRNA (pre-miRNA) in the nucleus by the Drosha enzyme. Afterward, they are exported to the cytoplasm by Ran-GTP and Exportin-5, where they are processed into mature microRNA (miRNA) with type III RNAse, Dicer (Figure 4) (Shirjang et al., 2019).

### 1.7. Breast cancer and microRNAs

miRNAs generally target multiple mRNAs. According to their expression or mutation levels, they can act as apoptomirs (supporting apoptosis), oncomirs (tumor promoters), or tumor suppressor miRNAs in different cancers. Since 2005, when miRNA dysregulation was first reported, there have been over 1000 studies identifying and examining the role of miRNAs in breast cancer. Due to the functional contribution of miRNAs in oncogenesis and their effect on the formation of chemoresistance, it is inevitable to consider them as molecular markers in the progression of resistance mechanisms to achieve high success in current chemotherapy applications (Shirjang et al., 2019).

Oncomirs (e.g., miR-21, miR-221/222) can induce cancer cell proliferation by reducing the expression of tumor suppressor genes. Whereas tumor suppressor miRNAs conversely target oncogenes. miRNA expressions differ according to the defined clinicopathological characteristics such as the stage of cancer, presence of cancer-triggering mutations, administered drug, patient’s survival process, and finally MDR. Mainly miR-34a, miR-221, miR-222, miR-10a, miR-31, miR-106b and miR-93, miR-29a, miR-30c, miR-125b, miR-21 are significantly upregulated in breast tumors, lead to poor prognosis, and are linked to drug resistance (Wu et al., 2014; Alamolhodaei et al., 2017; Ke and Lou, 2017; Lv et al., 2017; Li et al., 2017; Wu et al., 2019).

miR-21 has been shown as an indicator of clinical diagnostic approaches due to its ability to target proliferation signaling, tumor suppressor genes, metastasis and MDR-associated oncogenic function in HER2+BC. Overexpression of miR-21, advanced epithelial-mesenchymal transition (EMT) has been demonstrated in patients with poor prognosis (De Mattos-Arruda et al., 2015; Najjary et al., 2020). The use of aromatase inhibitors in postmenopausal HR+ patients are very common. miR 125b has been investigated among 90 patients of HR+ and metastatic BC to discover the molecular mechanisms underlying MDR progress. The results showed that the upregulation of AKT/mTOR pathway, overexpression of miR-125b or the silencing of miR-424 expression predispose the BC cells to acquire resistance to letrozole and anastrozole compared to control group (Vilquin et al., 2015; Zedain et al., 2020). The miRNA expressions screening of 246 tamoxifen-treated BC patients results indicated that miRNA-30c overexpression has a well prognosis outcomes, by comparison to the group with miRNA-30c low expression. The findings correlated the overexpression of miRNA-30c with the motility signaling pathways, and the stimulating growth factor receptor of this pathway, PDGFRa, was linked as a possible cause of tamoxifen resistance (Rodríguez-González et al., 2010; Han et al., 2020). Hereby, as a result of extensive research in this field, these small molecules are considered reliable biomarkers in BC diagnosis and alternative therapeutic approaches. 

### 1.8. Regulation of endoplasmic reticulum stress-activated apoptosis by microRNAs

**Figure 5:**
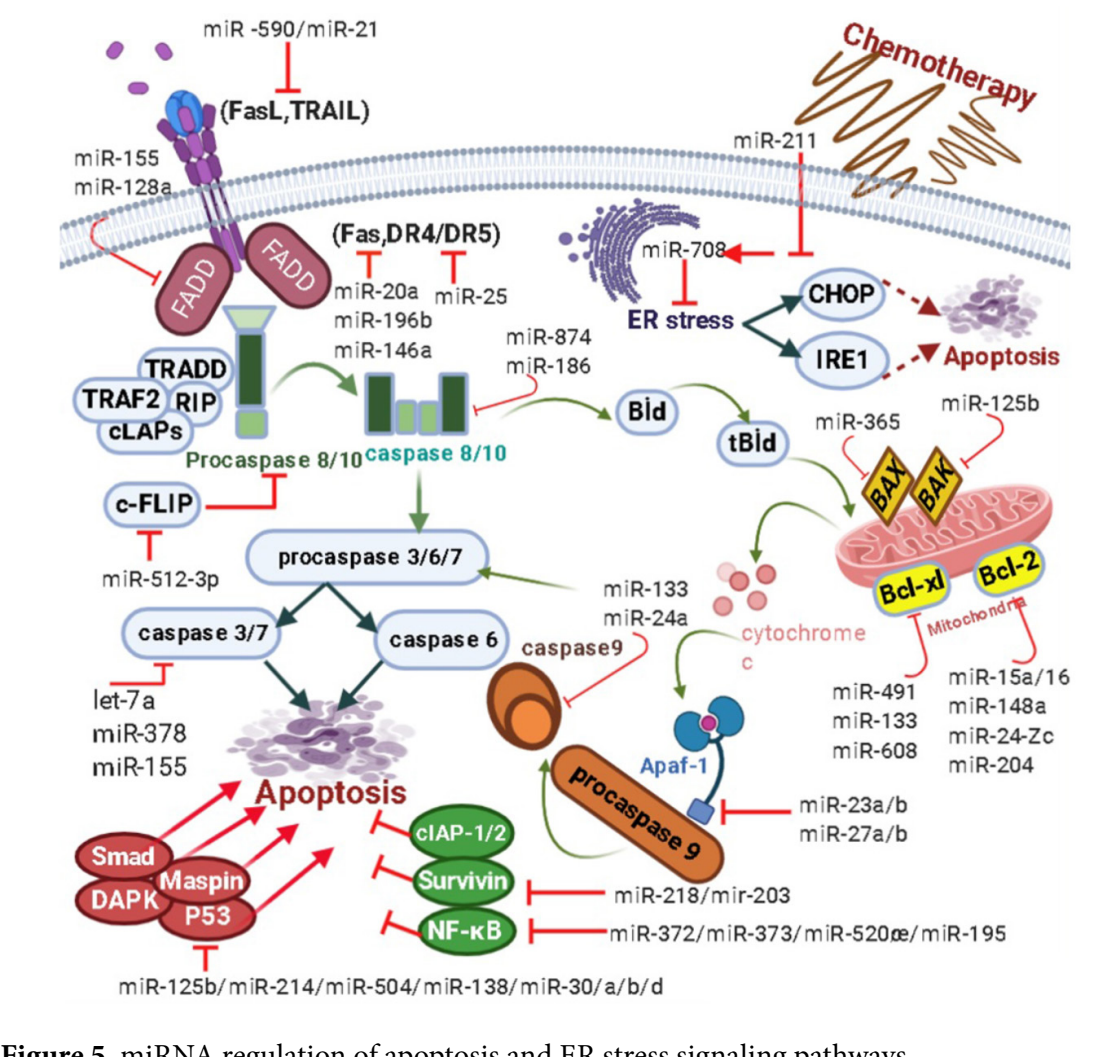
miRNA regulation of apoptosis and ER stress signaling pathways.

Posttranscriptional control, downstream of UPR, is one of the main important mechanisms in cancer development. This phenomenon is achieved through the selected mRNA expressions directly or transcriptional regulators such as miRNAs (Chevet et al., 2015). For example, miR-23a, miR-27a, miR-24-2 stimulates the expression of components such as CHOP, TRIB3, ATF3, and ATF4 in HEK293T cells, resulting in ER stress-induced apoptosis (Chhabra et al., 2011). To maintain tissue and organism homeostasis, damaged cells are abolished through programmed cell death, apoptosis, accompanied by miRNA surveillance. miRNAs are defined as apoptomir or oncomir in distinct tumors. While oncomirs support tumor growth, apoptomirs have been shown to control apoptotic pathways by targeting Bcl-2, Mcl-1, TRAIL, Fas, and p53 mRNAs (Shirjang et al., 2019). UPR’s three main receptors stimulate proapoptotic agents by performing CHOP activation, which is important for determining cell fate. It can urge apoptosis by targeting genes like GADD34 (growth arrest and DNA-damage-inducible 34), TRIB3, Bax, and Bcl-2. For example, the adapter protein, Src homology 2 domain containing 1 (SHC1), and the proapoptotic protein Bax are affected directly by overexpressed miR-365 leading to the occurrence of gemcitabine resistance in PDAC patients in invasive pancreatic ductal adenocarcinoma cells (PDAC). miR-125b provides resistance of breast cancer cells to paclitaxel by suppressing proapoptotic Bak expression. miR-491, by targeting Bcl-xL, has a role in inducing apoptosis, and significantly reduces the survival of human DLD-1 colorectal tumors. Also, miR-24-2c andmiR-148a reduce Bcl-2 expression. Moreover, miR-27a/b, miR-23a/b can control the sensitivity of neurons to apoptosis, as they act as endogenous inhibitors of APAF-1 expression. It has been reported that miR-24a and miR-133 directly inhibit caspase-9 to determine cell destiny. miR-211 induced by ER stress facilitates histone H3K27 methylation, resulting in the inductionof CHOP expression and cell survival. The expression level of miR-708 is enhanced by CHOP, which plays a major role in controlling ER stress and maintaining ER homeostasis (Figure 5) (Su et al., 2015).

## 2. Conclusion

Many chemical drugs preferred in clinical applications in cancer treatment, unfortunately, have a huge prevalence of traumatic side effects and MDR. Moreover, the existence of tumors at risk of becoming resistant to chemotherapy remains a serious obstacle to the chance of success in the treatment of various types of cancer. Indeed, many studies are being conducted on probable candidate therapeutic agents that have the potential to trigger other pathways to obtain alternative MDR modulators. The gene expression profile of cancer cells is a valuable tool in identifying new clinical biomarkers and molecular pathways underlying disease. Therefore, extensive research is needed to elucidate molecular mechanisms to develop effective targeted therapy options. The conflicting findings regarding the role of UPR, which is activated in cancer cells causing drug resistance, emphasize the need to understand the specific nature of UPR. Since the duration of ER stress is a determinant of cell fate, it iscritical to know how much the therapeutic dose obtained in tumor cells triggers ER stress.The fact that independent of the mechanism of action of drugs, ER stress, and UPR support cell survival or death should be considered as a determining factor.In conclusion, since UPR signaling mechanisms are active in breast cancer, it should be followed closely during the treatment processes of especially aggressive breast cancer patients in the clinic, because of its potential to promote the development and progression of the disease and to contribute to therapy resistance. Overall miRNAs act as modulators or effectors against UPR-associated sensors triggered by the ER stress, also the control of protein translation and ER entry, multidrug resistance development. However, by regulating target mRNAs in the progression of tumors, miRNAs show a suppressive and/or stimulating impact on gene expression and activation of prominent pathways. Studies have observed remarkable changes in the miRNA expression profile of resistant types in contrast to drug-sensitive cells. Up-to-date data obtained from the empirical studies mentioned in this review proves that master miRNAs target multiple basic signaling networks, tyrosine kinase receptor pathways, epigenetic suppressors, and transcription factors in tumors. It is also nominated as an MDR regulator by abnormally modulating the levels of various important gene expression modulators. Functional miRNAs as important candidate diagnosis and prognostic indicators of breast cancer are used in the development of new and more effective individualized therapies for cancer treatment. Hence the data obtained from the miRNA profile analysis of patients offer new opportunities for safer and faster diagnosis and planning heal processes.Numerous new techniques such as gene sequencing, proteomics, and microRNA analysis have been proposed to enhancement the survival procedure in cancer therapy.
